# SUR1 Receptor Interaction with Hesperidin and Linarin Predicts Possible Mechanisms of Action of *Valeriana officinalis* in Parkinson

**DOI:** 10.3389/fnagi.2016.00097

**Published:** 2016-05-02

**Authors:** Gesivaldo Santos, Lisandro Diego Giraldez-Alvarez, Marco Ávila-Rodriguez, Francisco Capani, Eduardo Galembeck, Aristóteles Gôes Neto, George E. Barreto, Bruno Andrade

**Affiliations:** ^1^Departamento de Ciências Biológicas, Universidade Estadual do Sudoeste da BahiaJequié, Brazil; ^2^Programa Nacional de Pós-Doutorado (PNPD-CAPES), Departamento de Química e Exatas, Universidade Estadual do Sudoeste da BahiaJequié, Brazil; ^3^Departamento de Nutrición y Bioquímica, Facultad de Ciencias, Pontificia Universidad JaverianaBogotá, DC, Colombia; ^4^Instituto de Investigaciones Cardiológicas “Prof. Dr. Alberto C. Taquini” (ININCA), UBA-CONICETBuenos Aires, Argentina; ^5^Departamento de Bioquímica, Instituto de Biologia, Universidade Estadual de Campinas—UNICAMPCampinas, São Paulo, Brazil; ^6^Departamento de Ciências Biológicas, Universidade Estadual de Feira de SantanaFeira de Santana, Brazil; ^7^Instituto de Ciencias Biomédicas, Universidad Autónoma de ChileSantiago, Chile; ^8^Universidad Científica del SurLima, Peru

**Keywords:** *Valeriana officinalis*, Parkinson disease, neuroprotection, SUR1, GABAA

## Abstract

Parkinson’s disease (PD) is one of the most common neurodegenerative disorders. A theoretical approach of our previous experiments reporting the cytoprotective effects of the *Valeriana officinalis* compounds extract for PD is suggested. In addiction to considering the PD as a result of mitochondrial metabolic imbalance and oxidative stress, such as in our previous *in vitro* model of rotenone, in the present manuscript we added a genomic approach to evaluate the possible underlying mechanisms of the effect of the plant extract. Microarray of substantia nigra (SN) genome obtained from Allen Brain Institute was analyzed using gene set enrichment analysis to build a network of hub genes implicated in PD. Proteins transcribed from hub genes and their ligands selected by search ensemble approach algorithm were subjected to molecular docking studies, as well as 20 ns Molecular Dynamics (MD) using a Molecular Mechanic Poison/Boltzman Surface Area (MMPBSA) protocol. Our results bring a new approach to *Valeriana officinalis* extract, and suggest that hesperidin, and probably linarin are able to relieve effects of oxidative stress during ATP depletion due to its ability to binding SUR1. In addition, the key role of valerenic acid and apigenin is possibly related to prevent cortical hyperexcitation by inducing neuronal cells from *SN* to release GABA on brain stem. Thus, under hyperexcitability, oxidative stress, asphyxia and/or ATP depletion, *Valeriana officinalis* may trigger different mechanisms to provide neuronal cell protection.

## Introduction

Parkinson’s Disease (PD) is the second most common progressive neurodegenerative disorder affecting older American adults and is predicted to increase in prevalence as the USA population ages. A population-based study of US Medicare beneficiaries found a mean prevalence of 1.6% for PD among persons 65 years and above (Beitz, 2014). This disease is characterized by a selective and progressive degeneration of dopaminergic neurons and the presence of Lewy bodies in the neurons of the Substantia Nigra pars compacta (SNpc; Cabezas et al., 2012, 2014; dos Santos et al., 2014, 2015; Willard et al., 2015). Imaging studies have shown that 70% of the dopaminergic neuron loss from SN is the result of a scarcity in the catecholamine neurotransmitter dopamine, generating the characteristic motor symptoms of PD (Rieckmann et al., 2015). The cause of dopaminergic cell death in PD remains unknown, but it seems like to be associated with a number of insults that may trigger programmed cell death such as calcium influx, oxygen free radicals species of oxygen (ROS) and mitochondrial complex I (Cx-I) inhibition (Hald and Lotharius, 2005; Abdin and Hamouda, 2008; Barreto et al., 2011; Cabezas et al., 2012).

Therapeutic efforts aimed at the removal of ROS or prevention of their formation may be beneficial in PD. In this regard, natural products are attractive sources of chemical structures that exhibit potent biological activities with desirable pharmacological profiles. Several reports have suggested that flavonoids and alkaloids may be useful to protect cells from ROS toxicity (Cummings and Zhong, 2006; Albarracin et al., 2012; Sutachan et al., 2012; Kwon et al., 2014; Bae et al., 2015; Hiep et al., 2015). The presence of flavonoids in *Valeriana officinalis* extract makes this plant attractive for the search of a daily neuroprotective product for human consumption (Adel Pilerood and Prakash, 2014). However, it is important to consider that an extract is a mixture of different substances and, thus, other compounds may be responsible for these effects. Candidates that can act in CNS may also include alkaloids, terpenes and phenolic compounds, volatile oils, like valerenic acid or valeranone, or the valepotriate group (Houghton, 1999; Kennedy and Wightman, 2011). In Brazil, *Valeriana officinalis* has been used in traditional medicine for its sedative, anticonvulsant, hypnotic effects, and anxiolytic activity (Carlini, 2003).

Previously, our group (de Oliveria et al., 2009) observed that *Valeriana officinalis* decreased rotenone-induced apoptosis in human neuroblastoma SH-SY5Y cells in a PD *in vitro* model. In spite of some hypothetical addressed designers and anatomic limitations in this model, the real mechanism involved in *Valeriana officinalis*-induced neuroprotection, not only *in vitro*, but also in a true systemic neural network on PD, remains to be completely delineated. The rotenone model largely only gained attention following the seminal article by the Greenamyre group, which demonstrated for the first time that rotenone administered systemically to rats can reproduce the hallmarks of PD (Betarbet et al., 2000).

Rotenone, a flavone pesticide, inhibits Cx-I of the mitochondrial electron transport chain leading to reduced ATP production and electron leakage that can form reactive oxygen species such as superoxide, subsequently causing reduced glutathione levels and oxidative stress (Cabezas et al., 2012; Johnson and Bobrovskaya, 2015). Some previous studies have shown that Cx-I impairment may be inherited in familial PD, indicating that Cx-I might account for some types of the sporadic forms of the disease (Dawson and Dawson, 2003). In this article, our aim was to provide some mechanistic insights into the neuroprotective action of the *Valerian’s* extract, taking into account the necessity of overcoming some clinical and structural constraints raised in our previous experimental model of PD in neuroblastome SH-SY5Y (de Oliveria et al., 2009). To accomplish this, we are using a powerful method of large-scale transcriptional profiling, combined with system biology techniques and pathway analysis to address the interactome of human *SN*, targeting genes related with the PD. Legal, ethical questions and high experimental costs make the computational system biology a reliable alternative as a back-end or first approach screening either before or after the laboratory bench (Edwards et al., 2011).

## Materials and Methods

In our previous study, de Oliveria et al. (2009), argued that the extract of *Valeriana officinalis* must be a target in the search for new agents for the treatment of PD. In this study, cell viability assays clearly showed that the aqueous extract of *Valeriana officinalis* protected SH-SY5Y cells against damage induced by rotenone, but the underlying mechanisms are not fully understood. Indeed, it is not clear how *Valeriana’s* extracts may act on human PD brain. To address this question we have to deal with two different issues: e.g., (i) why not all dopaminergic neurons fall in death in PD (Betarbet et al., 2000); and (ii) which of the extract components take part on this process. In spite of the structural constraint, we have performed a mechanistic model targeting changes in hub genes expression, assuming the PD as a result of imbalance between ROS and ATP depletion in mitochondria of neurons containing dopamine in SN (Yao and Wood, 2009).

Microarray data were retrieved from the Allen Brain Atlas^1^, an online database containing valued assets freely available of the entire genome of the human brain (Hawrylycz et al., 2012). Data were obtained interactively during the experiment, selecting the structures of interest from six subjects. The basal ganglia SN was chosen in a list, as being the most remarkable structure in PD. The array of genes obtained (Supplementary Table 1) were used as seed input list for the two applications Gene2Fan^2^ (Dannenfelser et al., 2012) and Expression2Kinase (Chen et al., 2012) tuning to developing network biology protein-protein interaction. To determine the high degree of connectivity between genes or “hub genes”, it was used the centrality measurement (CM). CM is related to the value function of a node in a graph representing a complex network. The index can be derived from the number of directed paths arriving or living the node. For better overview, see Opsahl et al. (2010).

**Figure 1:**
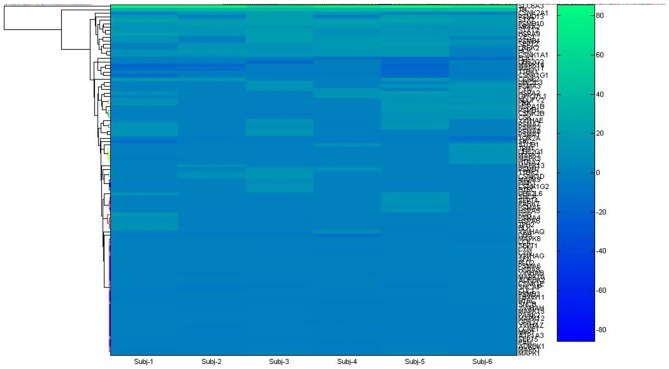
**Hierarchical clustering used to get insight about gene hub from microarray list**.

The next step was to access how the main components of *Valeriana* could be acting in the modulation of pathways associated with these transcripts on PD’s (Figure 1). In order to achieve this, we compared the similarity between the list of drugs obtained from X2Kinase (Chen et al., 2012) and Enricher^3^ (Chen et al., 2013), using the algorithm of similarity ensemble approach^4^ (Keiser et al., 2007). Similarity Ensemble Approach (SEA) makes use of the Tanimoto’s algorithm, a coefficient based in a reliable mathematical model for computing molecular similarities. Using this method, it is possible to generate a molecular fingerprint, which can be used to compare a set of multiple molecules at once in a reduced computing time. Different chemical groups are used to generate the fingerprint patterns (Bajusz et al., 2015).

The structure of each target molecule (transcripts of hub genes) was obtained from PDB.^5^ All *Valerian* compounds structures were obtained from PubChem database^6^ and saved in sdf format. In addition, ligand structures were previously checked by Marvin 15.4.20^7^ for docking studies. Before docking calculations, we used Autodock Tools 1.5.6 (Trott and Olson, 2010) in order to prepare protein and ligand structures according to the following steps: (1) define a grid box for each protein target, considering each active site previously described by crystallographic studies; (2) check each ligand structures for torsions, as well as adding polar hydrogens, Gaigster and Kollman charges; (3) save all molecules in pdbqt format. Autodock Vina (Trott and Olson, 2010) was used to calculate affinity energies for each complex. Discovery Studio 4.0 was used to generate 2D interaction maps, considering a 2.0 Å distance between the ligand and the active pocket amino acids. Molecular Dynamics (MD) simulations were performed by GROMACS 5.0.5^8^, using Molecular Mechanic Poison/Boltzman Surface Area (MMPBSA) protocol for 20 ns of simulation time. After each MD simulation set, we generated Root Mean Square Deviation (RMSD) graphics for both complexes and individual ligands, and for the number of hydrogen bonds generated during the simulation time.

## Results

**Figure 2:**
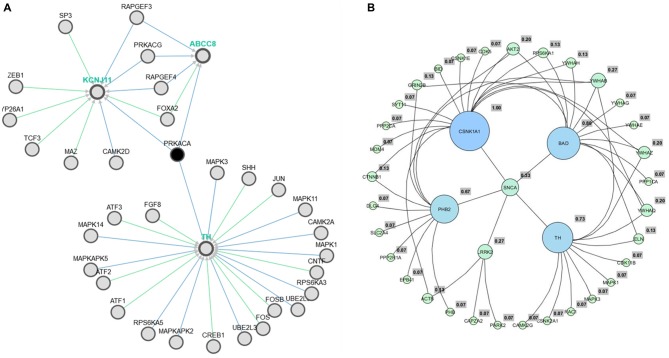
**Pathway association between TH and metabolic process via ABCC8 (Kir6.2) (A).** The six hub genes obtained from Allen Brain Atlas via yEd application **(B)**.

We have reconstructed a gene map of microarray expression from Allen Brain Atlas^9^, a public database maintained by Allen Brain Institute (Hawrylycz et al., 2012). In the first step, 101 genes (Supplementary Table 1) were organized in hierarchical clustering and used as seed input list of the applications Expression2Kinases (Chen et al., 2012) and Enricher^10^ to provide enrichment (GSEA; Chen et al., 2013). A list of 98 genes obtained was analyzed via yEd^11^ and optimized to genes associated to PD. Six clusters emerged forming the sub networks alpha-synuclein (SNCA), leucine-rich repeat kinase 2 (LRRK2), tyrosine hydroxylase (TH), Casein kinase 1 (CNSK1), Prohibitin 2 (PHB2) and Bcl-2-associated death promoter (BAD) (Figure 2A).

To assess genomic profile, the six hubs (Figure 2B) were confronted using AmiGO^12^ (Ashburner et al., 2000) and Enricher^13^ (Chen et al., 2013). As a result, all GO processes previously generated were related to proteasome, and PD. Data were confirmed from pathways resources such as Kegg^14^, BioCarta^15^ and Reactome^16^ assessed directly from Enricher^17^ (Chen et al., 2013).

**Figure 3:**
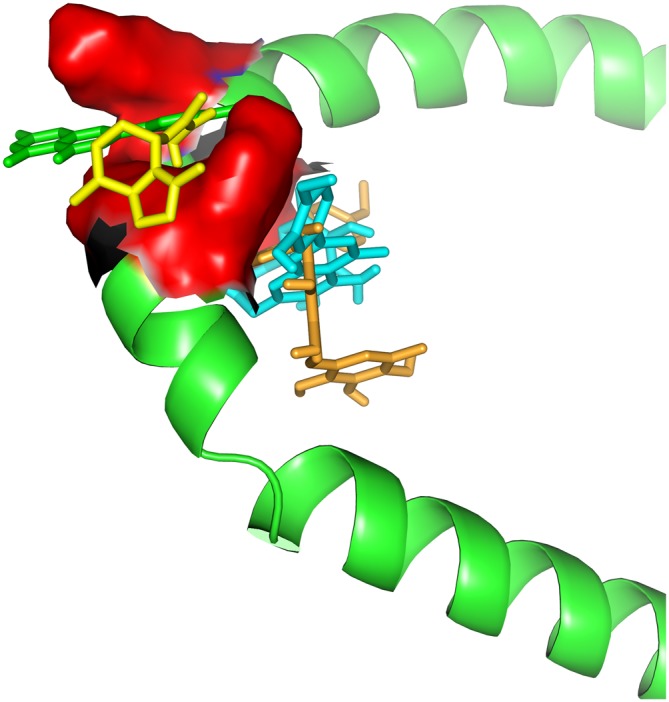
**Docking between alpha-synuclein (SNCA) and *Valerian* compounds.** Apigenin (green) and valerenic acid inside active site, while linarin and hesperidin appears out.

Even though the genome profile of SN has revealed six major genes, the use of centrality measure reported the gene SNCA as the highest degree. SNCA acts inhibiting TH directly, preventing dopamine release (Henchcliffe and Beal, 2008). To gain insights into these mechanism of action, we needed to filter out the genes, in these network, whose transcripts could have a potential involvement with dopamine, rotenone, mitochondrial metabolism, mitochondrial ROS imbalance and the compounds of *Valerian* related with the previous experiment of de Oliveria et al. (2009). But now, we have considered just a new structural parameter associated to a cortical inhibition by neuronal cells of *SN*. This process was achieved using Pathwaycommons, whose purpose was to identify commons pathways between hub genes.^18^ To perform molecular docking analysis (Figure 3) of transcripts from the same network linking SNCA and TH with the main compounds of the plant, we used Autodock Vina Tools (Trott and Olson, 2010; Table 1).

**Table 1:** **Affinity energies for docking calculations between alpha-synuclein and tyrosine hydroxylase with *Valerian* compounds**.

Target	*Valerian* compound	RMSD	Affinity energy (Kcal/mol)
SNCA	Apigenin	9.019	−5.1
	Valerenic acid	9.548	−4.4
	Valerenic acid	0	−7.4
TH	Apigenin	0	−8.9
	Linarin	0	−9.3
	Hesperidin	0	−9.4

To identify a viable mechanism of action for the *Valerian* extract, we selected apigenin, linarin, hesperidin and valerenic acid as the main representative compounds of the four families of substances present on the extract (National Toxicology Program, Chemical Information Review Document, 2009). These elements have been compared with drugs of recognized action on the SNCA and TH, both transcripts of the hub genes SNCA and TH, respectively (Table 2).

**Table 2:** **Similarity Ensemble Approach (SEA) search results for *Valerian* compounds**.

Known drug	*Valerian* compound	Max TC
Methylpredinisolone	Acid valenenic	0.60
Norethynodrel	Acid valenenic	0.59
Androsterone	Acid valenenic	0.52
Menadione	Apigenin	0.51
Atractyloside	Hesperidin	0.47
Atractyloside	Linarin	0.45
Resveratrol	Apigenin	0.45
Menadione	Linarin	0.42

### Ligand-Based Screening Approach

Drug targets were selected based on the profile of hub genes SNCA and TH generated by the Enricher application (Keiser et al., 2007). Furthermore, drugs were divided into cAMP up and down regulation (Table 2). The search tool SEA^19^ (Keiser et al., 2007), was used to retrieve the probable similarity between drugs and the targets of *Valerian*, using the Tanimoto’s Coefficient (Max TC; Keiser et al., 2009). According to Table 2, we observed that glucocorticoids are the major class of drug widely related to gene profile obtained for the SN. In SEA results, the main components of *Valerian* that shared structural similarity with target drugs was valerenic acid (Max TC = 0.60) and apigenin (Max TC = 0.51). Both key compounds of plant extract, which are recognized on literature as aggregation inhibitors of SNCA, as observed in Figure 3 (Uversky et al., 2014).

### Pathways and Molecular Docking

As observed in Figure 2A, obtained from the Pathwaycommons, the gene encoding the protein kinase-A (PRKCA), associated to several cell functions, such as apoptosis, emerges as a link between the hub gene SNCA, TH and the main mitochondrial genome expressed on SN cells.

**Figure 4:**
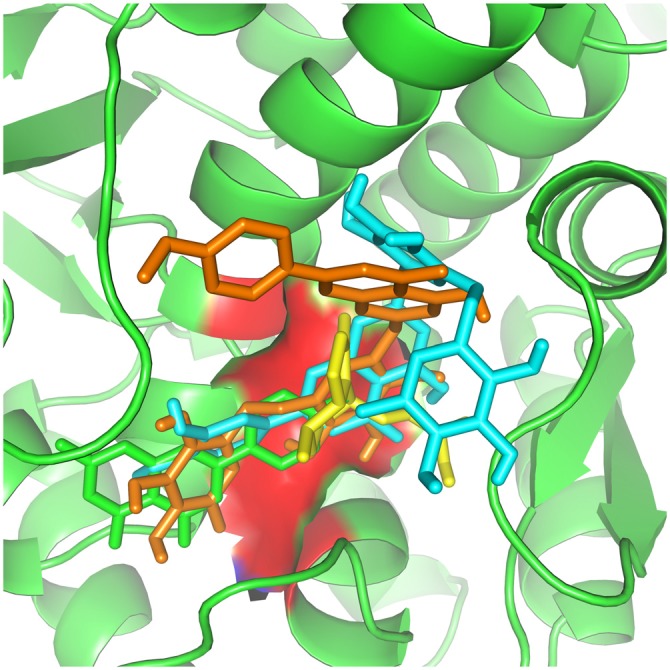
***Valerian* compounds inside TH active site.** In this docking, apigenin (green), valerenic acid (yellow), hesperidin (cyan) and linarin (orange), all the molecules interacting with TH active site.

**Figure 5:**
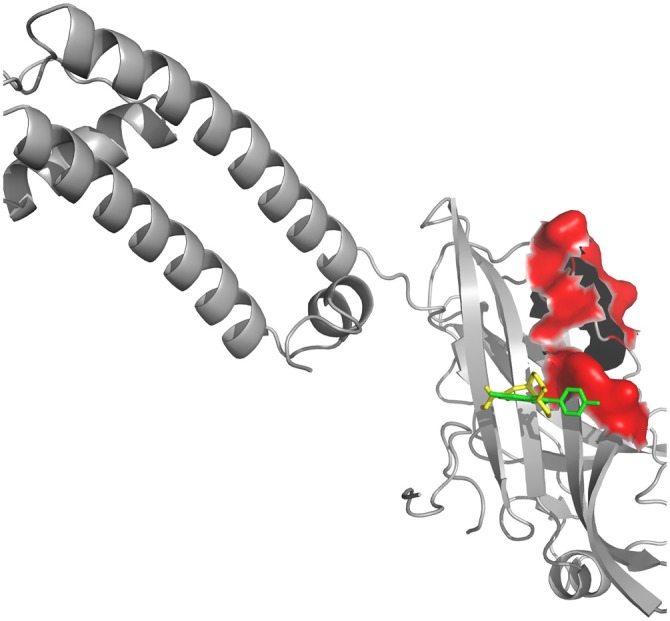
**Kir receptor complexed with *Valerian* compounds.** Apigenin (cyan) and valerenic acid (green) interacting with active site. Hesperidin and linarin did not fit in the binding site of Kir.

**Figure 6:**
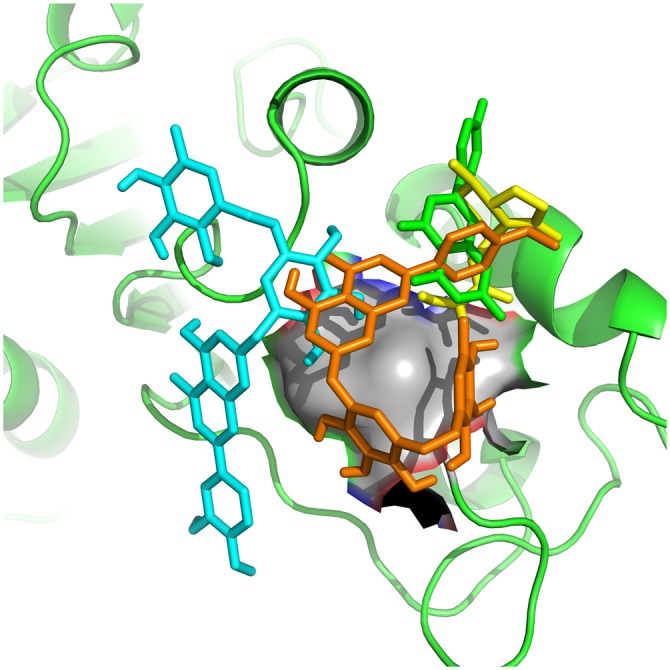
**Apigenin (cyan), valerenic acid (green), hesperidin (yellow) and linarin (orange) complexed with SUR1**.

**Figure 7:**
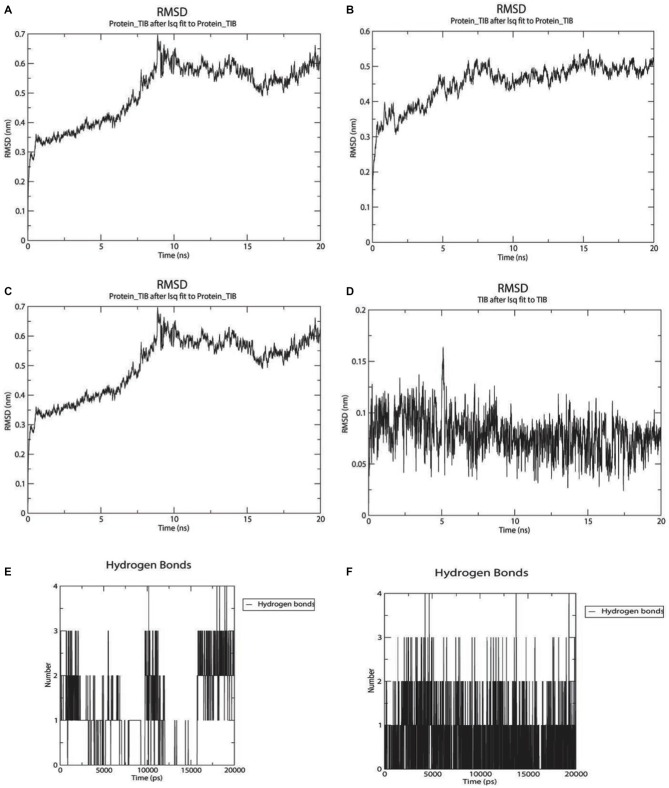
**Twenty nanoseconds Molecular Dynamics (MD) Molecular Mechanic Poison/Boltzman Surface Area (MMPBSA) for complexes between GABAA with apigenin and valerenic acid. (A)** Root mean square deviation (RMSD) fluctuations for GABAA-Apigenin complex; **(B)** RMSD fluctuations for valerenic acid; **(C)** Apigenin RMSD variation; **(D)** valerenic acid RMSD variations; **(E)** hydrogen bonds fluctuations for apigenin inside GABAA active site; **(F)** hydrogen bonds fluctuations for valerenic acid inside GABAA active site.

It appears significant that ABCC and KCNJ families emerge as a high connectivity node in this new subnetwork, since they are tied to metabolic control with the membrane potential of mitochondria (Akao et al., 2001; Deutch and Winder, 2006; Wang et al., 2011). Therefore, molecular docking was used to evaluate and quantify the interaction of *Valerian* compounds with TH, K^+^ inward rectifier channel (ABCC8) and sulfonylurea receptor (KCNJ11; Figures 4–6). According to the literature, GABAergic neuronal cells of Substantia Nigra pars compacta (SNpc), a remarkable area in PD, exhibit a large expression of ABCC8 to control GABA release on brain stem. In this way, we decided to dock GABAA receptor with components of *valerian* to predict whether the extract could act as a modulator of cortical excitability, feature not tested in de Oliveria et al. (2009), but capable of being evaluated in a *in silico* model hereafter. Dynamic simulations retrieve energy values for docking calculations between GABAA (2YOE), the target and the four *Valerian* compounds (ligands; Figures 7A–F).

### *Valerian* Compounds and GABA

**Figure 8:**
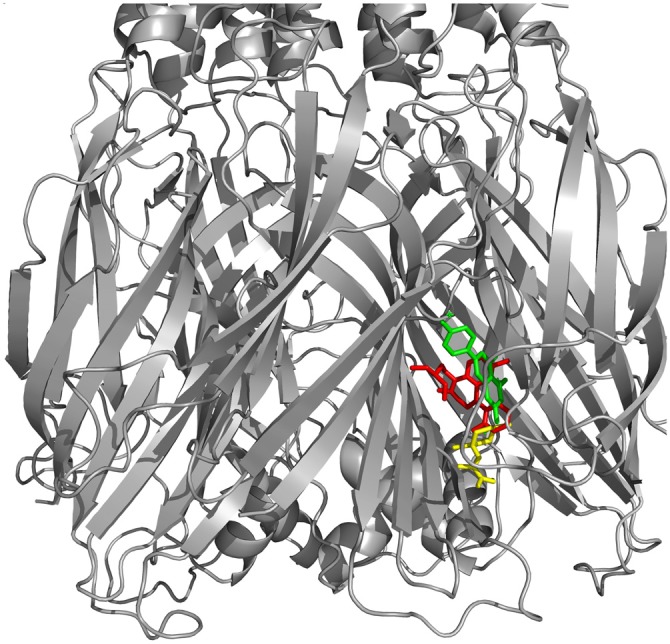
**GABAA receptor docked with Flurazepan and *Valerian* compounds.** Apigenin (yellow) and valerenic acid docked in the same site of Flurazepan (red).

As observed in Figures 7A–F, only valerenic acid and apigenin remain in the binding pocket after a set of 20 ns MD simulation. In order to validate the GABAergic effect of valerenic acid and apigenin with GABAA receptor (2YOE), we re-dock the crystallographic structure of Flurazepan (Figure 8), a standard ligand to this channel (Cope et al., 2005). MD simulation (Figures 7A–F, 8) showed complex behavior after 20 ns MMPBSA. The RMSD of 2YOE-valerenic acid complex was 0.5 Å, while in the complex 2YOE-apigenin was 0.6 Å. In the analysis of structural stability of both *Valeriana* ligands, valerenic acid presented an RMSD of 0.07 Å and apigenin, 1.3 Å. Furthermore, we observed constant fluctuations in GABAA, between 0.07 Å and 0.13 Å, when the receptor is complexed with apigenin. On the other hand, GABAA presented more stable when complexed with valerenic acid, ranging from 0.07 to 0.11 Å of fluctuation. Additionally, when we verified the number of hydrogen bonds, valerenic acid seemed more stable, changing from 2 to 3 hydrogen bonds, while apigenin presented just one hydrogen bond for all simulation time. As expected, *Valerian* compounds complexes in the same GABAA active site with Flurazepan, thus confirming our hypothesis (Figure 8).

### *Valerian* Compounds and SUR1

**Figure 9:**
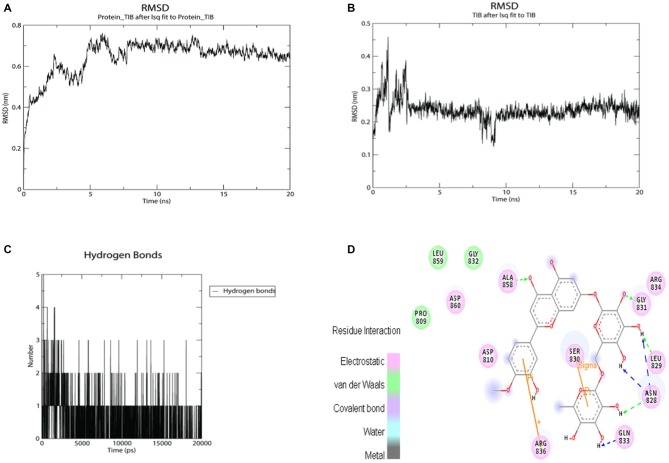
**MMPBSA simulation results for the complex SUR1 with hesperidin and 2D interaction map. (A)** RMSD fluctuations for SUR1-Hesperidin complex; **(B)** RMSD fluctuations for hesperidin; **(C)** hydrogen bonds fluctuations for hesperidin inside SUR1 active site; **(D)** 2D interaction map for hesperidin inside SUR1 active pocket.

Regarding the docking results (Table 3), as well as the 2D map interactions (Figure 9D), we selected hesperidin as the molecule with the best capacity to interact with SUR1. Figure 9A shows the RMSD fluctuations of the complex SUR1-hesperidin. As observed, the interaction was stable from the first steps of simulation until the end of the 20 ns. The RMSD of the ligand was almost invariable (Figure 9B), as well as the complex. From the beginning of the simulation up to 10 ns, the number of hydrogen bonds between SUR-hesperidin varied from 5 to 1 and after it reached three interactions at the end of the simulation (Figure 9C). The final binding free energy value at 20 ns was −119.771 KJ/mol (±4.841). These results indicated that SUR1 and hesperidin formed a strong and stable interaction from the beginning until the end of 20 ns of simulation. This can be explained because the SUR1 active site and hesperidin share most of hydrophobic features. Indeed, SUR1 may interact with Kir6.2, a transcript of ABBC8, thus forming a complete functional channel.

**Table 3:** **Affinity energies for docking calculations between Kir and SUR1 with *Valerian* compounds**.

Target	*Valerian* compound	RMSD	Affinity energy (Kcal/Mol)
SUR	Hesperidin	0	−6.3
	Linarin	0	−5.9
	Apigenin	0	−5.2
	Valerenic acid	0	−4.5
Kir	Apigenin	0	−6.5
	Valerenic acid	0	−5.5

## Discussion

PD includes multiscale dysfunctions associated to severe loss of nigrostriatal neurons mainly associated to oxidative stress attack, and abnormal concentration of amyloid plaques derived from the oxidation of substances such as dopamine and high amount of SNCA (Gaiteri et al., 2014). It is recognized that mutations in diferents genes such as SNCA, parkin (PRKN), LRRK2, PTEN-induced putative kinase 1 (PINK1) are involved in some sporadic forms of the PD (Wood-Kaczmar et al., 2006; Gaiteri et al., 2014). As reported by our analysis, only SNCA and TH are shown as a hub gene related to our previous experiments in de Oliveria et al. (2009) (Figure 2B). In spite of strong evidences, the precisely physiological function of SNCA and its interaction with TH on PD do not remain clear. However, some studies have shown that SNCA is differentially expressed in cell compartments. It has a high expression in inner mitochondrial membrane when compared to the cell cytoplasm. In mammalian brain, SNCA is predominantly expressed in hippocampal formation and striatum, with many evidences showing that the protein plays a role in several neurodegenerative disease (Dodson and Guo, 2007). A single mutation (SNP) of the SNCA gene can also lead to a general form of PD, resulting in the accumulation of Lewy bodies within the surviving cells (Mata et al., 2006). This reduction in solubility of synuclein modifies the action of the enzyme TH, resulting in an increased concentration of dopamine and cytoplasmic ROS generated from its derivatives in oxidation form (Qi et al., 2014). As observed in Table 1, SNCA does not seem to be the substrate of the main components of *Valeriana oficcinalis*. In contrast, TH and dopamine may be the key for the preferential susceptibility of death in neurons containing dopamine in PD. A great body of *in vitro* and *in vivo* evidences, especially those associated with pesticide rotenone and inhibition of Cx-I, have been very useful (Cabeza-Arvelaiz and Schiestl, 2012; Giordano et al., 2012). Nevertheless, if we consider that rotenone causes mitochondrial Cx-I inhibition on entirely brain regions, and not only on SN cells, these unspecified brain distribution and preference for neurons containing dopamine may suggest that inhibition of Cx-I should be a secondary route of toxicity. Therefore, there must be something intrinsically related to dopamine metabolism, such as ATP depletion, dopamine overproduction and imbalance of NAD+/NADH, which are capable to induce selective death of dopaminergic neurons (Cabeza-Arvelaiz and Schiestl, 2012). Furthermore, our experiments with molecular docking between rotenone and Cx-I revealed a lower affinity energy of −7.8 Kcal/mol when compared to −10 Kcal/mol (Supplementary Table 1) exhibited by the complex with NADH, putting forward a new consideration about rotenone and neuronal cell death.

Reduction on membrane mitochondrial potential (MMTP) decreases ATP/ADP concentrations, promoting Ca^++^ imbalance and increasing of ROS production as a consequence (Sai et al., 2008). Moreover, rotenone leads to upregulation and aggregation of SNCA and APP conversion into the form beta amiloid, which is related to endoplasmic reticulum (ER) stress by SNCA oligomer accumulation. These findings are in agreement with our analysis of centrality using microarray data (Figure 2B).

Impairments on mitochondrial respiration play several important functions in neuronal death on SN in PD, a cerebral region with dense expression of K-ATP channels (Henchcliffe and Beal, 2008; Perier and Vila, 2012).

The genes ABCC8 (SUR1), an ATP Bind Cassette subfamily, are associated to couple cellular energetic process to transmembrane potential by controlling the sensitivity of type ATP-inward-rectifier potassium channels, such that coded by kcnj4 and Kcnj11 (Liss et al., 2005). SN plays a key role in protecting neurons from seizures by preventing hyperexcitation under asphyxia or ATP depletion due to its capacity to release GABA in neuronal cell terminal. In this area, GABAergic pre-synaptic neurons have predominantly potassium ATP (KATP) channels that modulate the release of this neurotransmitter. An increase in ATP/ADP rates (high metabolic glucose consumption) closes the channel leading to cell depolarization by Ca^++^ influx (Liss et al., 2005; Sun and Feng, 2013). Under stress circumstances, such that induced by rotenone, the reduction of ATP/ADP prevents SN to release GABA, and, consequently, low levels of GABA may lead to cell damage as well as an increase in dopamine release (Amoroso et al., 1990; Sun and Feng, 2013).

According to our docking analysis (Figure 6), valerenic acid binds to SUR1 active site. This ligand accepts one hydrogen by its carboxyl group from ASN 828 and forms other electrostatic and Van der Waals interactions with other eight amino acids. These interactions are weak, when compared to the response of valerenic acid to GABAA receptors (Yuan et al., 2004; Khom et al., 2007, 2010; Trauner et al., 2008). In such situation, valerenic acid seems to have a secondary effect in SUR1 (Figure 6).

At the same way, docking between apigenin and SUR1 shows ARG 836 acting as a cation and forming two Pi interactions with the ligand. Other interactions are mainly electrostatic and the stereochemical effect is similar to valerenic acid, which explains its affinity with GABAA receptors (Avallone et al., 2000), reducing dopamine toxicity overproduction and preventing dopamine release from SN’s nerve cells, a pivotal area in development of PD.

Another possible neuroprotective effect exerted by *Valerian* in PD model reported by de Oliveria et al. (2009) appears to be accomplished by its flavones hesperidin and linarin on KATP channel sensor (SUR1). Complex hesperidin-SUR1 shows a strong interaction in the active site. ARG 836 and SER 830 formed Pi and Sigma interactions, respectively. The ligand accepted seven hydrogen interactions from GLN 833, ASN 828, LEU 829, GLY 831, ALA 858, and its stereochemistry seemed to make a more stabilized docking, in comparison to valerenic acid and apigenin. Moreover, hesperidin activity is described as reducing oxidative stress (Tirkey et al., 2005) and acting in MAPK pathway (Kim et al., 2011). At the same way of hesperidin, linarin fitted strongly to SUR1 receptor. The ligand mainly interacted to SER 857, accepting one hydrogen bond and donating two. Other interactions were electrostatic and Van der Waals.

As previously described, hesperidin and linarin are two of the main constituent of *Valeriana*’s extract exhibiting a high affinity to KATP channel, which are related to the control of Ca^++^ concentration and release of GABA in synaptic nerve terminal, mainly on cells of SN (Henchcliffe and Beal, 2008; Perier and Vila, 2012). Due to the blockage of KATP channel by binding to SUR1 (internal subunit associated to Kir6.2), flavones of *Valerian* should revert the membrane transient toward the positive status. At the same way, voltage sensitive Ca^++^ inward opens to restore the balance between ATP depletion. Theses flavones in such situation could resemble a ATP signaling, activating Kir6.2 to restore MMTP and thus preventing apoptosis in SN neuronal cell containing dopamine, a remarkable consequence in PD.

Our report brings a new approach using hesperidin and linarin relating to alleviating the effects of oxidative stress in PD. Some authors described linarin activity as sedative and sleep-enhancer (Fernández et al., 2004), but it was not related to any specific receptor. Additionally, linarin was described as a selective inhibitor of acetilcholinesterase (Oinonen et al., 2006). Thus, this is the first study describing the mechanism of interaction between SUR1 and linarin as a probable inhibitor ligand. Finally, in this work we suggest that hesperidin and linarin have a synergic activity in *Valerian’s* extracts, thus corroborating our previous experimental data in the *in vitro* PD model (de Oliveria et al., 2009).

## Conclusion

Despite the complexity relating to evaluating a plant extract and its constituent interactions, we performed several theoretical analysis trying to figure out the key role of the neuroprotective effect exerted by the extract of *Valeriana officinalis*. Its most remarkable effect is associated to anxiety and sleep modulation mediated by valerenic acid and the flavon apigenin by binding as agonists to GABAA receptors in brainstem (Trauner et al., 2008). Similarly in previous studies, our data suggest that there are, at least, two main pathways associated to pharmacological effect of *Valerian’s* neuroprotection in PD model (Riedel et al., 1982). It appears to be carried out by a GABAergic activity, reducing neuronal hyperactivity and dopamine release during oxidative stress, a situation associated to ATP/ADP mitochondrial balance.

## Author Contributions

All authors listed, have made substantial, direct and intellectual contribution to the work, and approved it for publication.

## Conflict of Interest Statement

The authors declare that the research was conducted in the absence of any commercial or financial relationships that could be construed as a potential conflict of interest.

## Footnotes

^1^www.brain-map.org/

^2^http://actin.pharm.mssm.edu/genes2FANs/

^3^http://amp.pharm.mssm.edu/Enrichr/

^4^http://sea.bkslab.org/search/

^5^www.pdb.org

^6^http://pubchem.ncbi.nlm.nih.gov/

^7^https://www.chemaxon.com/

^8^http://www.gromacs.org/

^9^http://www.brain-map.org

^10^http://amp.pharm.mssm.edu/Enrichr/

^11^www.yworks.com

^12^http://amigo.geneontology.org/amigo

^13^http://amp.pharm.mssm.edu/Enrichr/

^14^http://www.genome.jp/kegg/

^15^http://www.biocarta.com

^16^http://www.reactome.org/

^17^http://amp.pharm.mssm.edu/Enrichr/

^18^http://www.pathwaycommons.org

^19^http://sea.bkslab.org
