# Supplementary material for: SUR1 Receptor Interaction with Hesperidin and Linarin Predicts Possible Mechanisms of Action of Valeriana officinalis in Parkinson
Source: Front Aging Neurosci. 2016 May 2;8:97. doi: 10.3389/fnagi.2016.00097 (PMC4852538; doi:10.3389/fnagi.2016.00097)
Supplement: Supplementary file 1 [file DataSheet_1.docx]

| -0.6574 | 1059344 | A_24_P326635 | 153 | ADRBK1 | adrenergic, beta, receptor kinase 1 | |
| --- | --- | --- | --- | --- | --- | --- |
| 0.4365 | 1059340 | A_23_P251686 | 154 | ADRBK2 | adrenergic, beta, receptor kinase 2 | |
| -0.2560 | 1059001 | A_23_P27472 | 475 | ATP1A3 | ATPase, Na+/K+ transporting, alpha 3 polypeptide | |
| 0.0925 | 1058825 | A_23_P31725 | 637 | BLK | B lymphoid tyrosine kinase | |
| 0.6501 | 1024244 | A_23_P35427 | 8866 | BTRC | beta-transducin repeat containing E3 ubiquitin protein ligase | |
| 0.4953 | 1024243 | A_23_P46819 | 8866 | BTRC | beta-transducin repeat containing E3 ubiquitin protein ligase | |
| -0.1212 | 1058520 | A_23_P209200 | 893 | CCNE1 | cyclin E1 |  |
| 0.5887 | 1028578 | A_23_P215976 | 9011 | CCNE2 | cyclin E2 |  |
| 13.939 | 1018780 | A_23_P152024 | 1434 | CSK | c-src tyrosine kinase | |
| 0.8244 | 1026929 | A_24_P183292 | 1441 | CSNK1A1 | casein kinase 1, alpha 1 | |
| 0.1703 | 1023593 | CUST_8506_PI416261804 | 1442 | CSNK1D | casein kinase 1, delta | |
| 0.7481 | 1030575 | A_24_P918436 | 1443 | CSNK1E | casein kinase 1, epsilon | |
| -0.6932 | 1043021 | A_23_P14841 | 33239 | CSNK1G1 | casein kinase 1, gamma 1 | |
| 0.6628 | 1057901 | A_23_P56140 | 1444 | CSNK1G2 | casein kinase 1, gamma 2 | |
| 0.1567 | 1024302 | A_23_P502575 | 1446 | CSNK2A1 | casein kinase 2, alpha 1 polypeptide | |
| 12.135 | 1057897 | A_24_P804263 | 1449 | CSNK2B | casein kinase 2, beta polypeptide | |
| 0.1176 | 1049691 | A_23_P59657 | 8393 | CUL1 | cullin 1 |  |
| -0.8983 | 1057719 | A_23_P112296 | 1609 | DBH | dopamine beta-hydroxylase (dopamine beta-monooxygenase) | |
| -11.532 | 1012769 | A_23_P171054 | 1987 | ELK1 | ELK1, member of ETS oncogene family | |
| 0.2922 | 1045843 | A_23_P214046 | 23046 | FBXW11 | F-box and WD repeat domain containing 11 | |
| -0.3197 | 1021053 | A_24_P316634 | 2253 | FGR | Gardner-Rasheed feline sarcoma viral (v-fgr) oncogene homolog | |
| 11.465 | 1056884 | A_23_P133665 | 2428 | FRK | fyn-related kinase | |
| 13.409 | 1029625 | A_23_P502142 | 2518 | FYN | FYN oncogene related to SRC, FGR, YES | |
| -0.3228 | 1056226 | A_24_P85557 | 2844 | GPR37 | G protein-coupled receptor 37 (endothelin receptor type B-like) | |
| 12.954 | 1048769 | A_24_P145633 | 9152 | GPR37L1 | G protein-coupled receptor 37 like 1 | |
| 0.3019 | 1055751 | CUST_9876_PI416261804 | 3038 | HCK | hemopoietic cell kinase | |
| 16.616 | 1055439 | A_24_P123616 | 3284 | HSPA1B | heat shock 70kDa protein 1B | |
| 13.755 | 1055436 | A_23_P88303 | 3286 | HSPA2 | heat shock 70kDa protein 2 | |
| 0.5100 | 1055433 | A_24_P18190 | 3289 | HSPA5 | heat shock 70kDa protein 5 (glucose-regulated protein, 78kDa) | |
| 0.2517 | 1055431 | CUST_13369_PI416261804 | 3290 | HSPA6 | heat shock 70kDa protein 6 (HSP70B') | |
| 0.0711 | 1030129 | A_24_P287129 | 3292 | HSPA8 | heat shock 70kDa protein 8 | |
| 14.824 | 1055426 | A_24_P77676 | 3293 | HSPA9 | heat shock 70kDa protein 9 (mortalin) | |
| 13.475 | 1023398 | A_24_P336957 | 27173 | HTRA2 | HtrA serine peptidase 2 | |
| -0.0111 | 1021165 | A_23_P103361 | 3907 | LCK | lymphocyte-specific protein tyrosine kinase | |
| 0.9284 | 1022614 | A_23_P128447 | 78809 | LRRK2 | leucine-rich repeat kinase 2 | |
| 0.6353 | 1019123 | A_23_P147431 | 4042 | LYN | v-yes-1 Yamaguchi sarcoma viral related oncogene homolog | |
| -0.8911 | 1030603 | A_23_P257895 | 5562 | MAPK1 | mitogen-activated protein kinase 1 | |
| 0.6353 | 1019123 | A_23_P147431 | 4042 | LYN | v-yes-1 Yamaguchi sarcoma viral related oncogene homolog | |
| -0.8911 | 1030603 | A_23_P257895 | 5562 | MAPK1 | mitogen-activated protein kinase 1 | |
| 0.4829 | 1052658 | A_23_P45025 | 5570 | MAPK10 | mitogen-activated protein kinase 10 | |
| -0.7785 | 1052660 | A_23_P502274 | 5568 | MAPK11 | mitogen-activated protein kinase 11 | |
| -0.2662 | 1051816 | CUST_7613_PI416261804 | 6266 | MAPK12 | mitogen-activated protein kinase 12 | |
| -0.4840 | 1052657 | A_23_P145376 | 5571 | MAPK13 | mitogen-activated protein kinase 13 | |
| -0.9304 | 1057918 | A_23_P426292 | 1421 | MAPK14 | mitogen-activated protein kinase 14 | |
| -0.7730 | 1033033 | A_23_P60962 | 86280 | MAPK15 | mitogen-activated protein kinase 15 | |
| 11.986 | 1052670 | A_23_P37910 | 5563 | MAPK3 | mitogen-activated protein kinase 3 | |
| 11.872 | 1052662 | A_23_P100704 | 5566 | MAPK7 | mitogen-activated protein kinase 7 | |
| -0.9479 | 1030187 | A_23_P356152 | 5567 | MAPK8 | mitogen-activated protein kinase 8 | |
| -0.6799 | 1029475 | A_23_P167692 | 5569 | MAPK9 | mitogen-activated protein kinase 9 | |
| -0.4766 | 1054067 | A_23_P171296 | 4328 | MPP1 | membrane protein, palmitoylated 1, 55kDa | |
| 14.159 | 1053786 | A_23_P130418 | 4703 | NDUFV2 | NADH dehydrogenase (ubiquinone) flavoprotein 2, 24kDa | |
| 14.000 | 1053510 | A_23_P131208 | 4898 | NR4A2 | nuclear receptor subfamily 4, group A, member 2 | |
| -0.8031 | 1053372 | A_23_P2501 | 5021 | PAH | phenylalanine hydroxylase | |
| -0.5352 | 1026388 | A_23_P136077 | 5039 | PARK2 | parkinson protein 2, E3 ubiquitin protein ligase (parkin) | |
| 0.3511 | 1016251 | A_23_P74740 | 11151 | PARK7 | parkinson protein 7 | |
| 0.6938 | 1020910 | A_23_P23194 | 41542 | PINK1 | PTEN induced putative kinase 1 | |
| 0.4067 | 1052984 | A_23_P4308 | 5306 | PLD2 | phospholipase D2 | |
| 0.7181 | 1052574 | A_23_P150286 | 5650 | PSMA1 | proteasome (prosome, macropain) subunit, alpha type, 1 | |
| 0.7356 | 1052572 | A_23_P345591 | 5651 | PSMA2 | proteasome (prosome, macropain) subunit, alpha type, 2 | |
| 14.113 | 1030403 | A_23_P140301 | 5652 | PSMA3 | proteasome (prosome, macropain) subunit, alpha type, 3 | |
| 0.7891 | 1018209 | A_24_P124992 | 5653 | PSMA4 | proteasome (prosome, macropain) subunit, alpha type, 4 | |
| 0.7977 | 1052570 | A_23_P86216 | 5654 | PSMA5 | proteasome (prosome, macropain) subunit, alpha type, 5 | |
| -0.1921 | 1052568 | A_23_P25735 | 5655 | PSMA6 | proteasome (prosome, macropain) subunit, alpha type, 6 | |
| 0.6632 | 1052566 | A_23_P91468 | 5656 | PSMA7 | proteasome (prosome, macropain) subunit, alpha type, 7 | |
| 0.4774 | 1026780 | A_23_P367995 | 80313 | PSMA8 | proteasome (prosome, macropain) subunit, alpha type, 8 | |
| 13.120 | 1052564 | A_23_P156531 | 5657 | PSMB1 | proteasome (prosome, macropain) subunit, beta type, 1 | |
| 17.111 | 1052548 | A_23_P140807 | 5667 | PSMB10 | proteasome (prosome, macropain) subunit, beta type, 10 | |
| 0.0269 | 1052559 | A_23_P100576 | 5659 | PSMB3 | proteasome (prosome, macropain) subunit, beta type, 3 | |
| 11.308 | 1052557 | A_23_P761 | 5660 | PSMB4 | proteasome (prosome, macropain) subunit, beta type, 4 | |
| 0.8815 | 1052553 | A_23_P9362 | 5663 | PSMB7 | proteasome (prosome, macropain) subunit, beta type, 7 | |
| 16.535 | 1027337 | A_23_P75889 | 5687 | PSMD13 | proteasome (prosome, macropain) 26S subunit, non-ATPase, 13 | |
| 0.4946 | 1057627 | A_23_P21057 | 1719 | SEPT1 | septin 1 |  |
| 10.543 | 1026097 | A_23_P10839 | 4709 | SEPT2 | septin 2 |  |
| 0.9306 | 1052917 | A_23_P207003 | 5382 | SEPT4 | septin 4 |  |
| -0.6124 | 1052921 | A_23_P17724 | 5381 | SEPT5 | septin 5 |  |
| 0.3867 | 1056293 | A_23_P63254 | 2793 | SFN | stratifin |  |
| 73.706 | 1051500 | A_24_P397435 | 6495 | SLC6A3 | solute carrier family 6 (neurotransmitter transporter, dopamine), member 3 | |
| 0.9969 | 1020182 | A_23_P29939 | 6582 | SNCA | synuclein, alpha (non A4 component of amyloid precursor) | |
| -0.1921 | 1021916 | A_23_P19020 | 9483 | SNCAIP | synuclein, alpha interacting protein | |
| -0.6367 | 1051280 | A_23_P121945 | 6580 | SNCB | synuclein, beta | |
| -0.0715 | 1030191 | A_23_P308603 | 6674 | SRC | v-src sarcoma (Schmidt-Ruppin A-2) viral oncogene homolog (avian) | |
| 13.813 | 1047607 | A_23_P15073 | 10127 | STUB1 | STIP1 homology and U-box containing protein 1, E3 ubiquitin protein ligase | |
| 12.108 | 1045444 | A_23_P385206 | 23426 | STX12 | syntaxin 12 | |
| 17.195 | 1049725 | A_23_P303260 | 8358 | STX7 | syntaxin 7 |  |
| 39.239 | 1026629 | A_23_P258633 | 7012 | TH | tyrosine hydroxylase | |
| -13.544 | 1044437 | A_24_P192556 | 27177 | TOR2A | torsin family 2, member A | |
| 15.858 | 1050661 | A_23_P150595 | 7123 | TPH1 | tryptophan hydroxylase 1 | |
| -0.2768 | 1035831 | A_24_P129834 | 78832 | TPH2 | tryptophan hydroxylase 2 | |
| -0.1507 | 1024256 | CUST_1292_PI416379584 | 58829 | TTBK1 | tau tubulin kinase 1 | |
| 0.3314 | 1034702 | A_24_P224342 | 80493 | TTBK2 | tau tubulin kinase 2 | |
| 11.064 | 1050498 | A_23_P27066 | 7283 | UBE2G1 | ubiquitin-conjugating enzyme E2G 1 | |
| 0.1041 | 1025757 | A_23_P211179 | 7284 | UBE2G2 | ubiquitin-conjugating enzyme E2G 2 | |
| 12.453 | 1050494 | A_23_P166353 | 7289 | UBE2L3 | ubiquitin-conjugating enzyme E2L 3 | |
| 0.7270 | 1030542 | A_23_P75741 | 9120 | UBE2L6 | ubiquitin-conjugating enzyme E2L 6 | |
| 0.2839 | 1050303 | A_23_P164507 | 7482 | YES1 | v-yes-1 Yamaguchi sarcoma viral oncogene homolog 1 | |
| 0.1068 | 1050298 | A_23_P120414 | 7486 | YWHAB | tyrosine 3-monooxygenase/tryptophan 5-monooxygenase activation protein, beta polypeptide | |
| -0.3563 | 1013580 | A_24_P123011 | 7488 | YWHAE | tyrosine 3-monooxygenase/tryptophan 5-monooxygenase activation protein, epsilon polypeptide | |
| 0.2540 | 1050296 | A_24_P106681 | 7489 | YWHAG | tyrosine 3-monooxygenase/tryptophan 5-monooxygenase activation protein, gamma polypeptide | |
| -0.6994 | 1050294 | A_23_P103070 | 7490 | YWHAH | tyrosine 3-monooxygenase/tryptophan 5-monooxygenase activation protein, eta polypeptide | |
| 0.4335 | 1046736 | A_32_P202057 | 10813 | YWHAQ | tyrosine 3-monooxygenase/tryptophan 5-monooxygenase activation protein, theta polypeptide | |
| -0.1172 | 1017602 | A_24_P209571 | 7491 | YWHAZ | tyrosine 3-monooxygenase/tryptophan 5-monooxygenase activation protein, zeta polypeptide | |
