# Supplementary material for: SUR1 Receptor Interaction with Hesperidin and Linarin Predicts Possible Mechanisms of Action of Valeriana officinalis in Parkinson
Source: Front Aging Neurosci. 2016 May 2;8:97. doi: 10.3389/fnagi.2016.00097 (PMC4852538; doi:10.3389/fnagi.2016.00097)
Supplement: Supplementary file 2 [file Table_1.pdf]

Table 1

Molecular docking studies for Complex I							
Rotenone				NADH+H			
mode	Affinity (Kcal/Mol)	Dist. from rmsd	Best mode rmsd	mode	Affinity (Kcal/Mol)	Dist. from rmsd	Best mode rmsd
1	-7.3	0.000	0.000	1	-10.1	0.000	0.000
2	-7.1	30.779	33.815	2	-9.6	1.500	2.582
3	-6.8	2.137	4.055	3	-7.6	7.627	11.490
4	-6.7	2.707	10.625	4	-7.5	31.685	36.289
5	-6.7	1.861	2.452	5	-7.4	31.721	36.214
6	-6.6	1.880	9.458	6	-7.4	27.032	32.157
7	-6.6	2.744	10.189	7	-7.3	27.076	31.999
8	-6.5	2.421	9.875	8	-7.2	33.154	36.793
9	-6.4	3.991	9.532	9	-7.2	27.659	33.147

Molecular docking calculations for the interaction beetween Mitochondrial Complex I with Rotenone and NADH+H. Table shows different docking poses and affinities for each molecular ligand's conformations.
